# Comparison of Perioperative Ranibizumab Injections for Diabetic Macular Edema in Patients Undergoing Cataract Surgery

**DOI:** 10.1155/2016/7945619

**Published:** 2016-07-14

**Authors:** Erhan Yumuşak, Kemal Örnek

**Affiliations:** Department of Ophthalmology, Kırıkkale University Faculty of Medicine, 71450 Kırıkkale, Turkey

## Abstract

*Purpose*. To compare the efficacy of perioperative ranibizumab injections on diabetic macular edema (DME) in patients undergoing cataract surgery.* Methods*. This study included 59 eyes of 59 patients. All patients had advanced cataract with DME and underwent an uneventful phacoemulsification surgery. There were 3 subgroups. The first group received intravitreal ranibizumab injection 2 weeks preoperatively, the second group received intraoperatively, and the third group received 2 weeks postoperatively. Follow-up examinations were performed at 1 week as well as at 1 and 3 months.* Results*. Baseline visual acuity showed a significant increase in all groups at 1 month. In group 1, compared to baseline value, foveal thickness (FT) increased significantly at 1 month and showed a significant decrease up to month 3. In group 2, FT increased at month 1 and this continued up to month 3. In group 3, FT increased at month 1 and was almost stable up to month 3. There were not any significant differences for visual acuity and FT between the groups.* Conclusions*. Although intrapostoperative ranibizumab injection for DME seems to be more effective than preoperative injections in patients undergoing cataract surgery, the treatment still needs to be continued following surgery.

## 1. Introduction

Both diabetic macular edema (DME) and cataract are common causes of decreased vision in patients with diabetic retinopathy (DR). Sometimes effective macular laser photocoagulation may be difficult or not possible to perform in some patients with DME when there is a coexisting cataract. Diabetes mellitus (DM) has been associated with an increased risk of postoperative ME. The incidence of ME on optical coherence tomography (OCT) is 22% in patients with diabetes undergoing cataract surgery [1, 2].

Patel et al. reported that vascular endothelial growth factor (VEGF) levels in the aqueous humor increased as early as the first day following cataract surgery and normalized by 1 month in patients with diabetes [3]. Studies in this field have been conducted using different anti-VEGF agents such as pegaptanib sodium, bevacizumab, and ranibizumab (RAN) [4, 5]. Ranibizumab is a human monoclonal antibody that inhibits all VEGF-A isoforms, and a detailed analysis of phase 3 clinical trials has generated evidence-based guidelines for using RAN for the treatment of DME [6].

Krohne et al. measured intraocular pharmacokinetics of RAN, looking in particular at concentrations in human aqueous samples, and found an aqueous half-life of 7.19 days [7]. In addition, Muether et al. used Luminex technology to measure VEGF suppression in the aqueous humor and showed that the therapeutic effect disappeared after 33.7 ± 5.1 days when they analyzed samples obtained during cataract surgery [8]. Prophylaxis of DME after cataract surgery with intravitreal RAN in patients with DR has been shown in previous studies, although, owing to the short half-life, the effect is transient and could show individual changes [9, 10].

In this study, we aimed to compare the efficacy of perioperative intravitreal ranibizumab injections for DME in patients undergoing cataract surgery.

## 2. Methods

This prospective clinical study included 59 eyes of 59 patients. It was conducted between 2014 and 2015 in accordance with the principles of the Declaration of Helsinki. The trial protocol has been approved by the Medical Ethical Committee of the University of Kırıkkale.

Inclusion criteria were sight-limiting cataract with a poor view of the fundus precluding adequate monitoring and/or macular laser treatment and the presence of DME determined by fluoresce in angiography (FA) and spectral domain optical coherence tomography (SD-OCT).

Exclusion criteria were presence of active intraocular inflammation, intractable glaucoma, age-related macular degeneration, epiretinal membrane, past ocular trauma or intraocular surgery, and intravitreal drug injection and/or retinal laser treatment within the previous 6 months.

At initial visit, ophthalmological examination included measurement of best corrected visual acuity (BCVA) which was measured with the Snellen chart and then converted into* Logarithm* of the Minimum Angle of Resolution (Log MAR), axial length, refractive status (spherical equivalent), and intraocular pressure (IOP) using Goldmann applanation tonometry and slit lamp examination. Fundus changes were assessed using SD-OCT (Retina Scan Advanced RS-3000, NIDEK, Gamagori, Japan) and FA (Canon CF-1 Digital Mydriatic Retinal Camera, Canon Inc., Tokyo, Japan). Cystoid pattern involving the central zone in OCT and FA imaging was defined as DME.

There were 3 subgroups. The first group (*n* = 20) received an intravitreal RAN injection 2 weeks preoperatively, the second group (*n* = 23) received an intraoperative injection, and the third group (*n* = 16) received an intravitreal RAN injection 2 weeks after surgery. All phacoemulsification cataract surgeries were performed under topical anesthesia by a single surgeon. A solution (0.05 mL) containing 0.5 mg of RAN was injected intravitreally under sterile conditions. After surgery, follow-up examinations were performed at 1 week, 1 month, and 3 months. A complete ophthalmic examination and SD-OCT imaging were performed at each visit.

SPSS 18.0 was used for statistical analyses (SPSS, Inc., Chicago, IL). The Pearson correlation test was used to evaluate relationship between variables. Parametric differences between the groups were assessed using a multivariate analysis. Paired *t*-tests were used to compare continuous variables with Bonferroni correction. A *p* value below 0.05 or 0.0167 with Bonferroni correction was regarded as statistically significant.

## 3. Results

Fifty-nine (59 eyes) patients who underwent phacoemulsification cataract surgery with intravitreal injection of 0.5 mg of RAN were included in this study. There were 36 female and 23 male patients. The mean age of the patients was 62.1 ± 5.65 years (range: 51–83 years). All patients (100%) had nonproliferative DR with cystoid-type DME preoperatively.  Table 1 shows the demographics of the study groups.

Group 1 included 20 patients with a mean hemoglobinA_1c_ (HbA_1c_) value of 8.77% ±  %1.29, (median: 8.22%, range: 8.0%–11.0%), including 8 women (40%) and 12 men (60%); mean age was 60.9 ± 1.16 years (median: 60.50, range: 60–63). Mean axial length was 22.47 ± 0.82 mm and mean refraction was (spheric equivalent) −0.10 ± 0.66 diopters (D).

There were 23 patients in group 2. The mean HbA_1c_ value was 9.04% ± 1.13%, (median: 8.95%, range: 8.0%–12.0%) and this group comprised 18 women (78.26%) and 5 men (21.74%) with a mean age of 64.8 ± 8.5 years (median: 65, range: 51–83). In this group, mean axial length was measured as 22.89 ± 0.57 mm with a mean refraction of −0.14 ± 0.44 D.

Group 3 had 16 patients with a mean HbA_1c_ value of 9.26% ± 1.37% (median: 9.28%, range: 6.9%–11.3%), including 10 females (62.5%) and 6 males (37.5%). The mean age was 61.0 ± 5.61 years (median: 61, range: 57–76). Axial length was 22.81 ± 0.74 mm and mean refraction was −0.18 ± 0.70 D.

**Figure 1:**
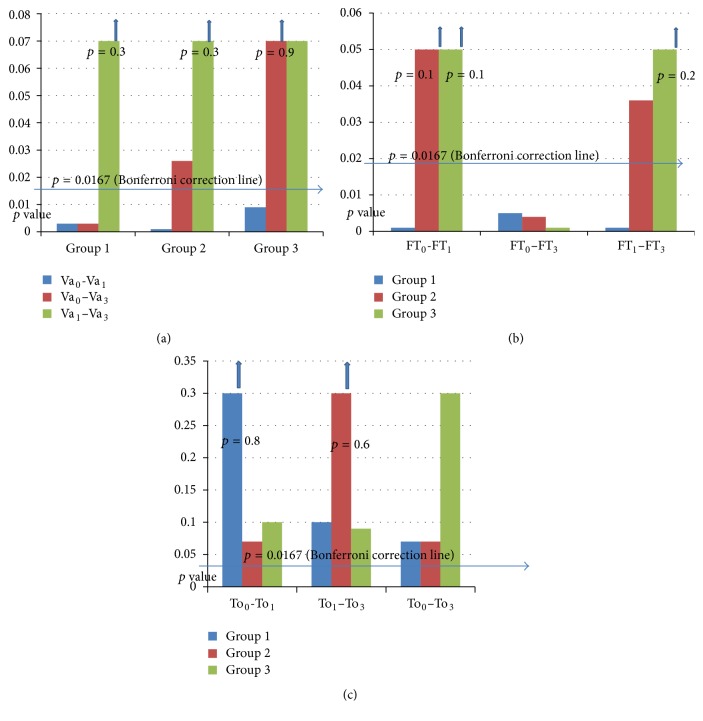
Comparisons between pre- and postoperative (a) BCVA, (b) FT, and (c) IOP changes in study groups (*t*-test with Bonferroni correction). Va_0_ = best corrected visual acuity at baseline, Va_1_ = best corrected visual acuity at 1st month, Va_3_ = best corrected visual acuity at 3rd month, FT_0_ = foveal thickness at baseline, FT_1_ = foveal thickness at 1st month, FT_3_ = foveal thickness at 3rd month, To_0_ = intraocular pressure at baseline, To_1_ = intraocular pressure at 1st month, and To_3_ = intraocular pressure at 3rd month.

Between the 3 groups, there were not any significant differences in terms of age, refractive status, axial length measurements, and HbA_1c_ values (all *p* > 0.05). No significant differences between pre- and postoperative IOP were observed (*p* > 0.05). Baseline BCVA showed a significant increase in all groups at 1 month (all *p* < 0.05). It was still significant in group 1 at month 3 (*p* < 0.05) (Figure 1).  Table 2 shows comparisons between pre- and postoperative BCVA, FT, and IOP changes in the study groups.

**Figure 2:**
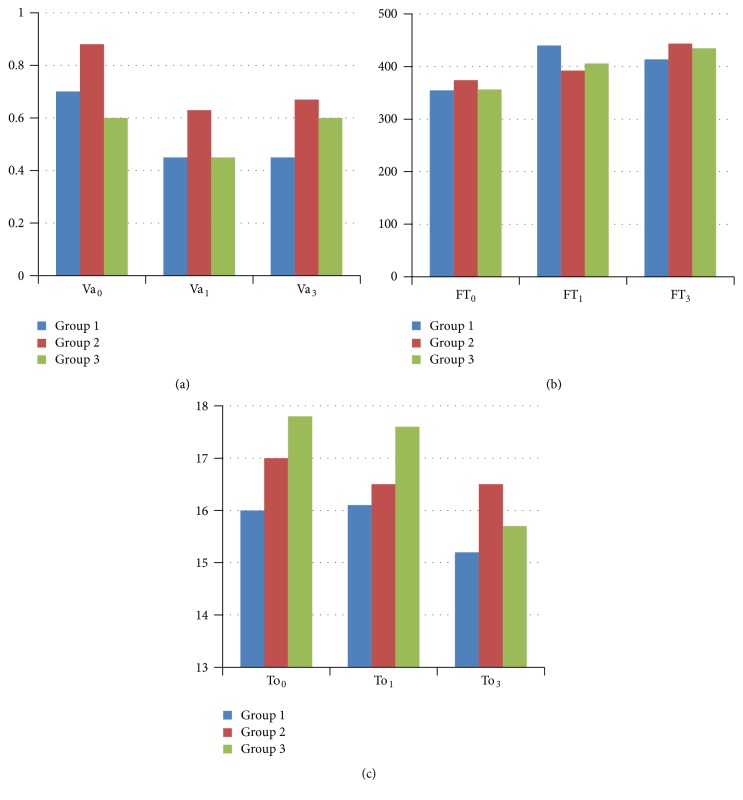
Changes of clinical parameters during the follow-up in study groups. (a) Best corrected visual acuity. (b) Foveal thickness. (c) Intraocular pressure. Va_0_ = best corrected visual acuity at baseline (Log MAR), Va_1_ = best corrected visual acuity at 1st month (Log MAR), Va_3_ = best corrected visual acuity at 3rd month (Log MAR), FT_0_ = foveal thickness at baseline, FT_1_ = foveal thickness at 1st month, FT_3_ = foveal thickness at 3rd month, To_0_ = intraocular pressure at baseline, To_1_ = intraocular pressure at 1st month, and To_3_ = intraocular pressure at 3rd month.

*Intragroup Comparisons*. In group 1, compared to baseline value (354.5 *μ*), foveal thickness (FT) increased significantly at 1 month (439.7 *μ*) (*p* < 0.0167) and showed an insignificant decrease up to month 3 (413.4 *μ*) (*p* < 0.0167). In group 2, FT almost slightly increased at month 1 (374.1 *μ* versus 392.0 *μ*) (*p* > 0.0167) and this continued up to month 3 (443.4 *μ*) (*p* < 0.0167). In group 3, FT increased insignificantly at month 1 (356.1 *μ* versus 406.0 *μ*) (*p* > 0.0167) and was stable up to month 3 (434.6 *μ*) (*p* > 0.0167) (Table 3, Figure 2).

*Intergroup Comparisons*. When we compared BCVA, FT, and IOP among three groups at baseline and 1 and 3 months after cataract surgery, there were not any significant differences for Va_0_, Va_1_, Va_3_, FT_0_, FT_1_, and FT_3_ between the 3 groups (all *p* > 0.05) (Table 4).

There was a negative correlation between BCVA and FT at baseline and 1 and 3 months after cataract surgery (*p* < 0.001, *r* = −0.595; *p* = 0.003, *r* = −0.379; and *p* < 0.001, *r* = −0.610, resp.).

No intraoperative complications (posterior capsular rupture, vitreous loss, and dropped lens fragments) or postoperative complications (endophthalmitis, retinal tears, and retinal detachment) were observed.

## 4. Discussion

There is an increased incidence of postoperative macular edema in eyes with DR [2, 11]. Macular edema after cataract surgery in diabetic patients may be the consequence of cataract surgery, existing diabetic retinopathy, or both, but it is typically difficult to differentiate between the two causes.

Diabetic macular edema results from multiple biochemical and cellular changes that eventually lead to vascular leakage and exudation. Increased permeability factors such as interleukin-6 and VEGF and an impaired blood-retina barrier lead to the passage of intravascular fluid into the intraretinal and subretinal spaces through microaneurysms and abnormal capillaries observed in this disease [12]. The Early Treatment of Diabetic Retinopathy Study (ETDRS) showed a significant benefit of focal macular laser photocoagulation for eyes with clinically significant ME (CSME) [1].

In an effort to improve anatomic and visual outcomes in the treatment of DME with grid laser photocoagulation, investigators have sought other treatment modalities like intravitreal injection of corticosteroids [13, 14]. Corticosteroids are proposed to decrease intraocular inflammation, cell proliferation, and neovascularization. By controlling the inflammation and inhibiting the expression of vascular growth factors, they may improve the integrity of the blood-retina barrier; however, intravitreal steroids have some disadvantages, including intractable steroid-induced glaucoma and cataract formation [15]. To avoid transient antiedema effect of steroids, slow releasing intravitreal steroids like dexamethasone implant were also developed. However, though its long acting effect, this new medication did not prevent secondary glaucoma and cataract [16].

Anti-VEGF drugs have a potential of treating coexisting DME in cataract patients undergoing surgery. Patel et al. measured VEGF levels in 7 diabetic patients after cataract surgery. There was a 10-fold increase at postoperative day 1, and, by the end of first month, VEGF levels showed a significant reduction (2.5-fold) [3]. Krohne et al. found a half-life of 7.19 days for RAN in human aqueous humor and Muether et al. reported a therapeutic effect lasting 33.7 ± 5.1 days for intraocular RAN [7, 8].

Chen et al. reported significant visual improvement and FT reduction after intraoperative bevacizumab injection in 15 patients [17]. Akinci et al. found similar results, but they added grid laser photocoagulation at the first postoperative month [18]. Opposite to these results, Cheema et al. did not observe any significant improvement in FT and VA during their 6 months of follow-up [19]. Though widely used all over the world, bevacizumab is still an off-label drug for the treatment of DME.

The effect of RAN on DME in patients undergoing cataract surgery has been shown in a small number of studies. Rauen et al. used RAN intraoperatively in 11 refractory DME patients undergoing cataract surgery; however, there was no control group in their study [20]. The authors reported that BCVA improved at 4, 8, and 12 weeks following cataract extraction. Six patients additionally received macular laser photocoagulation due to increased FT at 4 weeks. They did not find any significant difference in FT postoperatively and linked this to the refractory nature of the ME. Chae et al. investigated the effect of intraoperative RAN following cataract surgery in 76 DR patients without ME. They had 39 patients in the phacoemulsification group and 37 patients served as controls. There was no significant difference in FT or BCVA postoperatively at 6 months. Only total macular volume significantly differed in RAN patients [9].

In the present study, we compared pre-, intra-, and postoperative RAN injections for DME in patients undergoing cataract surgery. Visual acuity significantly improved in three injection groups. Foveal thickness increased in all groups after surgery, although this increase was less marked in patients who were treated with intra- and postoperative RAN injection. In a previous study, VEGF levels showed a significant reduction by the end of the first month following cataract surgery and also it was found that half-life of RAN in human eye is 7.19 days [7]. These data may explain the differences between preoperative injection and intra- and postoperative injections. A shorter interval between preoperative injection and cataract surgery may improve the outcomes. There were no laser treatments during the follow-up period. Although intergroup comparisons did not show any significant differences for BCVA and FT, intragroup analysis provided benefit for intraoperative injection of RAN in DME patients.

Intravitreal RAN injection does not seem to have a significant effect on postoperative ME following cataract surgery in DR patients. We propose that a single injection of RAN is perhaps insufficient to treat coexisting DME following cataract surgery and repeat injections may be required during the follow-ups. There may also be additional factors such as inflammation in these eyes to consider other than VEGF inhibition. Therefore, combination therapies (anti-VEGF agents and nonsteroidal anti-inflammatory drugs and steroids and laser photocoagulation) may be an option in DME patients undergoing cataract surgery. A common approach to these patients is first stabilizing the retinopathy and then planning the cataract surgery; this may help to prevent DME in patients with diabetes.

To conclude, in this study, intraoperative injection of RAN was found to be more effective than pre- and postoperative injection for DME. The results also confirmed that ongoing ranibizumab treatment is needed for most patients with DME. Two significant limitations of this study were small sample size due to strict inclusion criteria and lack of control group without intravitreal RAN injection which also do not allow us to make specific treatment recommendations. Further studies with longer follow-up and larger groups are needed to confirm the efficacy of RAN in the treatment of DME in patients undergoing cataract surgery.

## Figures and Tables

**Table 1:** Demographic values of the participants in each subgroup.

Group	*N*	L (R/L)	G (M/L)	Age^*∗*^	HbA_1c_
1	20	10/10	12/8	60.9 ± 1.16	8.77 ± 1.29
2	23	14/9	5/18	64.0 ± 8.38	9.04 ± 1.13
3	16	12/4	6/10	62.8 ± 2.55	9.26 ± 1.37

Total	59	36/23	23/36	62.1 ± 5.65	9.01 ± 1.34

*∗* indicates years.

L = laterality, R = right eye, L = left eye, G = gender, M = male, and F = female.

**Table 2:** Comparisons between pre- and postoperative BCVA, FT, and IOP changes in study groups (*t*-test) (*p* = 0.05/3 = 0.0167, Bonferroni correction).

Group	Va_0_-Va_1_	Va_0_–Va_3_	Va_1_–Va_3_	FT_0_-FT_1_	FT_0_–FT_3_	FT_1_–FT_3_	To_0_-To_1_	To_1_–To_3_	To_0_–To_3_
1	0.003	0.003	0.3	<0.001	0.005	<0.001	0.8	0.1	0.07
2	<0.001	0.026	0.3	0.1	0.004	0.036	0.07	0.6	0.07
3	0.009	0.9	0.07	0.1	<0.001	0.2	0.1	0.09	0.3

Va_0_ = best corrected visual acuity at baseline, Va_1_ = best corrected visual acuity at 1st month, Va_3_ = best corrected visual acuity at 3rd month, FT_0_ = foveal thickness at baseline, FT_1_ = foveal thickness at 1st month, FT_3_ = foveal thickness at 3rd month, To_0_ = intraocular pressure at baseline, To_1_ = intraocular pressure at 1st month, and To_3_ = intraocular pressure at 3rd month.

**Table 3:** Changes of clinical parameters^*∗*^ during the follow-up in study groups.

	Va_0_	Va_1_	Va_3_	FT_0_	FT_1_	FT_3_	To_0_	To_1_	To_3_
1	0.70 ± 0.79	0.45 ± 0.43	0.45 ± 0.43	354.5 ± 87.7	439.7 ± 113.7	413.3 ± 110.6	16,0 ± 4.6	16,1 ± 4.6	15,2 ± 3.0
2	0.88 ± 0.88	0.63 ± 0.72	0.67 ± 0.69	374.1 ± 81.7	392.0 ± 74.2	443.4 ± 126.2	17,0 ± 2.6	16,5 ± 1.6	16,5 ± 1.2
3	0.60 ± 0.65	0.45 ± 0.53	0.60 ± 0.74	356.1 ± 117.4	406.0 ± 129.7	434.6 ± 97.4	17,8 ± 2.2	17,6 ± 4.1	15,7 ± 1.9

^*∗*^Mean-standard deviation.

Va_0_ = best corrected visual acuity at baseline (Log MAR), Va_1_ = best corrected visual acuity at 1st month (Log MAR), Va_3_ = best corrected visual acuity at 3rd month (Log MAR), FT_0_ = foveal thickness at baseline, FT_1_ = foveal thickness at 1st month, FT_3_ = foveal thickness at 3rd month, To_0_ = intraocular pressure at baseline, To_1_ = intraocular pressure at 1st month, and To_3_ = intraocular pressure at 3rd month.

**Table 4:** *p* values of intergroup multicomparisons (multivariate test).

Groups	Age	Va_0_	Va_1_	Va_3_	FT_0_	FT_1_	FT_3_	To_0_	To_1_	To_3_	HbA_1c_
1-2	0.2	0.5	0.09	0.3	0.8	0.3	0.7	0.7	0.9	0.9	0.8
1–3	1.0	0.7	0.1	0.6	1.0	0.7	0.9	0.3	0.6	0.8	0.6
2-3	0.2	0.6	0.07	0.8	0.9	0.9	0.9	0.6	0.1	0.9	0.9

Va_0_ = best corrected visual acuity at baseline, Va_1_ = best corrected visual acuity at 1st month, Va_3_ = best corrected visual acuity at 3rd month, FT_0_ = foveal thickness at baseline, FT_1_ = foveal thickness at 1st month, FT_3_ = foveal thickness at 3rd month, To_0_ = intraocular pressure at baseline, To_1_ = intraocular pressure at 1st month, and To_3_ = intraocular pressure at 3rd month.
